# Psychometric Validation of the Indonesian Version of Children’s Revised Impact of Event Scale 13

**DOI:** 10.3390/ijerph192417069

**Published:** 2022-12-19

**Authors:** Okki Dhona Laksmita, Min-Huey Chung, Yann-Yann Shieh, Pi-Chen Chang

**Affiliations:** 1 School of Nursing, College of Nursing, Taipei Medical University, Taipei 11031, Taiwan; 2 Department of Nursing, Shuang Ho Hospital, Taipei Medical University, New Taipei City 23561, Taiwan; 3 Office of Special Education and Rehabilitation Service, U.S. Department of Education, Washington, DC 20202-2800, USA

**Keywords:** adolescent, CRIES-13, posttraumatic stress disorder, psychometric properties, scale validation

## Abstract

A psychometric evaluation of the Children’s Revised Impact of Event Scale 13 (CRIES-13), which measures the posttraumatic stress disorder (PTSD) symptoms in children and adolescents caused by the coronavirus disease 2019 (COVID-19) pandemic, was conducted. We aimed to cross-culturally adapt and test the psychometric qualities of the CRIES-13 that was applied to Indonesian adolescents exposed to COVID-19 risk across gender groups. A cross-sectional study was conducted at a junior high school in Yogyakarta, Indonesia, in May 2022; 346 Indonesian adolescents aged 12 to 18 years completed the Indonesian version of the CRIES-13. The factorial validity results supported the scale’s three-factor structure (intrusion, avoidance, and arousal), which satisfied all parameter index requirements and exhibited a favorable level of internal consistency reliability. Excellent results were obtained across genders for the four-level measurement of invariance (i.e., configural, metric, scalar, and full invariance), and they met the recommended criteria. Our Cronbach’s alphas and composite reliability ratings were high (>0.7), indicating a strong correlation and reliability of the items for measuring each construct. We strongly support the use of the Indonesian CRIES-13, which was demonstrated to be valid and reliable for an adolescent population.

## 1. Introduction

The COVID-19 pandemic has been characterized as a multisystem cascading disaster because it affects multiple systems and levels of response, ranging from the individual to the community and from the local to the national levels [1,2]. From January 2020 to December 2022, Indonesia was one of the most affected Southeast Asian countries, recording 6,674,000 confirmed COVID-19 cases and 159,921 deaths [3]. The percentages of these confirmed cases and deaths among those aged 18 years or younger are 13.8% and 1.2%, respectively [4]. The pandemic has been a traumatic event, with negative effects on the mental health of vulnerable populations such as children and adolescents [5], potentially leading to their development of posttraumatic stress disorder (PTSD) [6]. The development of COVID-19-related PTSD symptoms in children and adolescents has been discussed [7] and widely observed [8,9,10,11,12,13]. PTSD triggers a set of psychological symptoms that include re-experiencing, avoidance of event-related stimuli, negative changes in beliefs and feelings, and hyperarousal [14], all of which, if left untreated, can cause considerable distress and result in the development of functional disabilities [15].

The early detection of PTSD symptoms in children and adolescents is crucial to ensure that those who require additional evaluation and/or treatment can receive them in a timely manner [16]. However, a common problem that hinders early detection is the absence of accessible screening scales. The effective screening scales for PTSD require resources that are difficult or impossible to access in Indonesia. Furthermore, no information regarding psychometric characteristics has been provided in studies that used the scales for Indonesian children and adolescents, making it impossible to assess whether the scales are suitable for such use. Psychometric characteristics are essential evidence for researchers to determine the validity of a scale. Therefore, the accessibility of low-cost, thoroughly verified metrics that have been translated into Indonesian and evaluated in Indonesia must be enhanced.

The Children’s Revised Impact of Event Scale 13 (CRIES-13) is a straightforward, child-friendly measure that is theoretically supported and enables the valid and reliable screening of children and adolescents at risk for PTSD [17]. Studies have psychometrically tested the scale in various settings (e.g., traumatic events). The scale’s original three-factor structure was verified to be valid for children and adolescents who experienced natural and manmade disasters in Bangladesh [18], children and adolescents who experienced earthquakes in China [19,20] and Greece [21], clinical and normative samples from Republic of Korea [22], and children and adolescents who experienced military violence in Palestine [23]. However, studies have also reported inconsistent structural patterns, such as a two-factor structure among Chinese children and adolescents exposed to debris flood [24] and Portuguese children and adolescents exposed to wildfires [25], indicating the necessity of assessing these patterns in the present study. Additionally, few studies have examined the scale in the context of the COVID-19 pandemic, and no study has conducted a psychometric validation of the scale in the context of the Indonesian population. The effective screening of children and adolescents at risk of exposure to COVID-19 for PTSD is crucial to the development of future intervention strategies to enable children and adolescents to recover from the negative effects of the pandemic.

Gender differences in CRIES-13 results have been observed [18,23,25]. The tendency to report distress was higher among girls than among boys [26], and factor structures appeared to be different between genders [27]. Therefore, scale invariance must be verified to determine whether the conceptualization of a given measure is similar between genders. All facets of PTSD symptoms should be accurately measured using a valid and reliable scale. We evaluated the CRIES-13 by using it to assess PTSD symptoms in Indonesian adolescents of both genders at risk of exposure to COVID-19.

## 2. Materials and Methods

### 2.1. Study Design, Setting, and Participants

In March 2022, we conducted a cross-sectional study at a junior high school in Yogyakarta City, Indonesia. The school was randomly selected, and adolescent students were enrolled via purposive sampling after obtaining consent from the school’s principal and teachers. The sample size for this study was calculated on the basis of confirmatory factor analysis (CFA), the rule of thumb and model complexity rules for structural equation modeling [28], and the ratio of cases to free parameters in our model [29]. On the basis of those considerations and the 13 items of the CRIES-13, the sample size required to obtain robust estimations through the hypothesized model was estimated to be 260–500.

After obtaining consent to perform the research from the school’s principal and verbal consent from the school’s teachers, we obtained consent from the participating students’ parents or guardians through an online consent form. In addition, assent was obtained from students through an online assent form. Students were assured that all information collected would be anonymized. Student were enrolled if they were aged 12–18 years, had parental consent, and completed the online assent form. Students with COVID-19 during the study period were excluded.

### 2.2. Instrument

The CRIES-13 was developed by Smith et al. [17] on the basis of the Impact of Events Scale (IES). The IES was originally designed by Horowitz et al. [30] to monitor the main phenomena of a traumatic event (i.e., re-experiencing, avoidance, and feelings) in Western adult populations. The CRIES-13 is a short, self-reported measure that is designed specifically for children and written in language appropriate for all children with a reading age of at least 8 years. It has three subscales: intrusion (items 1, 4, 8, and 9), avoidance (items 2, 6, 7, and 10), and arousal (items 3, 5, 11, 12, and 13). All items are rated on a 4-point Likert scale with the following points: *not at all* (0), *rarely* (1), *sometimes* (3), and *often* (5). The total score ranges from 0 to 65, with a higher score indicating a greater likelihood of experiencing posttraumatic symptoms [17]. The scale exhibits satisfactory internal consistency; its Cronbach alphas for intrusion, avoidance, arousal, and overall were 0.70, 0.73, 0.60, and 0.80, respectively. A study revealed a three-factor solution corresponding to the three hypothesized subscales, with the solution accounting for 49.3% of the total variance [17].

### 2.3. Translation of CRIES-13

Permission to adapt the CRIES-13 was obtained from the original authors [17]. We applied Beaton’s translation and adaption methodology [31] and carefully documented all translation and adaption steps. First, two forward translations of the original English version of the scale into the Indonesian language were performed independently by two translators who were native Indonesian speakers. The first translator had experience in performing disaster nursing research and had knowledge of the PTSD concept; therefore, she was competent at achieving translation equivalence with respect to the translation of the instrument. By contrast, the second translator, who did not have a medical or clinical background and was not informed of the concept being explored, provided ideas that reflected the general language used by Indonesian people and highlighted any ambiguous content in the original measure. The two Indonesian translations of the CRIES-13 were then synthesized and compiled into a single version by the two translators and the researchers.

In the next step, the compiled Indonesian translation was independently back-translated into English by two translators who were native English speakers. These two translators did not have medical or clinical backgrounds and were not informed of the concept being explored. The purpose of this process was to ensure same-item consistency with the original instrument, check validity, and identify inconsistencies or conceptual errors in the translation.

Next, we convened an expert committee comprising all four translators, a methodologist, a language professional, and a mental healthcare professional to coordinate, review, and verify all versions of the scales (original, forward-translated, and back-translated and their corresponding written reports) and make a prefinal decision regarding field testing. Semantic, idiomatic, experiential, and conceptual equivalences were the key components considered during this process [31]. When questions and discrepancies could not be addressed, preceding steps were repeated as required. Only sentences that lost their original meaning were back-translated and retranslated.

We pilot tested the scale by using it to assess PTSD in 42 adolescents aged 12–18 years (24 students from a junior high school and 18 students from a senior high school) from a district of Yogyakarta. We asked participants to indicate whether the instructions, response structure, and components of the scale were clear; we also asked participants to provide suggestions on how the scale could be modified to increase its clarity if they found it to be unclear. The results from this process indicated that a brief explanation about the meaning of unclear items should be added.

### 2.4. Statistical Analysis

#### 2.4.1. Descriptive Statistics

IBM SPSS (Windows version 24) software (IBM Corp., Armonk, NY, USA) was used to analyze data pertaining to the demographic characteristics of the participants and the main study variables. Demographic characteristics examined were age, gender, grade level, school, religion, and living arrangement. Means (standard deviations [SDs]) and numbers (percentages) were used to describe the study variables.

#### 2.4.2. Content Validity Index

We convened an expert panel consisting of six Indonesian professionals who were experts in the fields corresponding to the scale’s constructs. We assessed the scale’s content validity to appraise the relevance and representativeness of each item with respect to its corresponding domain [32]. We adopted a systematic procedure for quantifying content validity indices (CVIs) that included estimating an item-level CVI (I-CVI), a scale-level CVI (S-CVI), an S-CVI based on averaging (S-CVI/Ave), and an S-CVI based on universal agreement (S-CVI/UA) [32,33].

Each expert was requested to appraise each item on a 4-point rating scale with the following points: *irrelevant* (1), *somewhat relevant* (2), *quite relevant* (3), and *highly relevant* (4). Items rated as 3 or 4 were converted to valid (1), and items rated as 1 or 2 were converted to nonvalid (0). The average and individual item ratings for each item were considered, and a UA was included to facilitate the evaluation of interrater reliability. An I-CVI score was calculated by dividing the number of experts who gave a rating of valid (1) by the total number of experts. An S-CVI/Ave score was calculated by dividing the sum of I-CVI scores by the total number of items. An S-CVI/UA score was calculated by dividing the number of items with 100% agreement by the total number of items [33]. Because more than five experts participated in the appraisal, the lower limit for acceptable I-CVI and S-CVI/Ave scores was set to 0.80. Items with an I-CVI score of < 0.80 were revised [33], and items with a score of < 0.70 should be eliminated [34]; the scale’s S-CVI/Ave was subsequently readjusted using the scores of the remaining items [33].

#### 2.4.3. CFA

The hypothesized model was developed using IBM SPSS Analysis of Moment Structures 23.0 software [35]. The evaluation of the model was based on several parameters. First, factor loading, which indicates the correlation between a variable (item) and a factor (construct), was appraised using the following cutoff values: poor (0.32), fair (0.45), good (0.55), very good (0.63), and excellent (0.71) [36,37]. Our model only included items with a standardized factor loading of > 0.45 and a significant *p* value (*p* < 0.05). Second, the goodness of fit of the model was determined by applying the following criteria: a Tucker–Lewis index (TLI) of > 0.90, a comparative fit index (CFI) of > 0.90, a root mean square error of approximation (RMSEA) of < 0.08, and a standardized root mean square residual (SRMR) of < 0.08 [38,39]. Additionally, the model was evaluated using modification indices (MIs) if goodness of fit was not achieved. A modification involving the addition of parameters (e.g., parameters derived from the residual covariances of identified items) was considered when MI > 10 to provide a strong theoretical foundation and enhance the fit of the model [40,41].

#### 2.4.4. Measurement Invariance Testing: Multigroup CFA

Multigroup CFA (MGCFA) is an extension of traditional CFA. Instead of fitting the data set with a single model, we divided our data set by gender (male and female), determined the model fit for each group independently, and performed multigroup comparisons. Through this method, we determined whether the participants from the two gender groups conceptualized a given metric similarly [42].

Four levels of measurement invariance were applied hierarchically for this analysis: configural (all parameters including factor loadings and item intercepts were left open for variation, but the structural model was held constant), metric (the factor loadings were set to be equal across gender groups, whereas the item intercepts were set to vary freely), scalar (factor loadings and item intercepts were set to be constrained), and full invariance (all parameters including factor loadings, intercepts, error variances, factor variances, covariances, and factor means were constrained) [42]. If the difference between two nested models (based on ΔCFI and ΔRMSEA) was < 0.01, measurement invariance was achieved between the gender groups [43,44]. Hierarchically arranged invariances result in an MGCFA terminating at the lowest level of invariance that cannot be satisfied [43].

#### 2.4.5. Reliability

A reliability test was conducted to assess the consistency of the responses to the scale items. Each factor’s internal consistency was calculated on the basis of the overall sample and each gender group’s Cronbach’s alpha and composite reliability, for which a cutoff point of >0.70 for acceptable values was applied [45,46].

## 3. Results

### 3.1. Participant Characteristics

A total of 350 participants completed the scale assessment; however, only the data collected from 346 were eligible for analysis. No missing data were detected with respect to these 346 participants. Participant demographic characteristics are listed in Table 1.

### 3.2. CVI

None of the CRIES-13 items required revision because the I-CVI of the 13 items ranged from 0.83 to 1, indicating item-level content validity. The S-CVI/Ave and S-CVI/UA for the CRIES-13 were 0.99 and 0.92, respectively, validating the content of the CRIES-13 with respect to the Indonesian adolescent population (Table S1).

### 3.3. CFA

**Figure 1:**
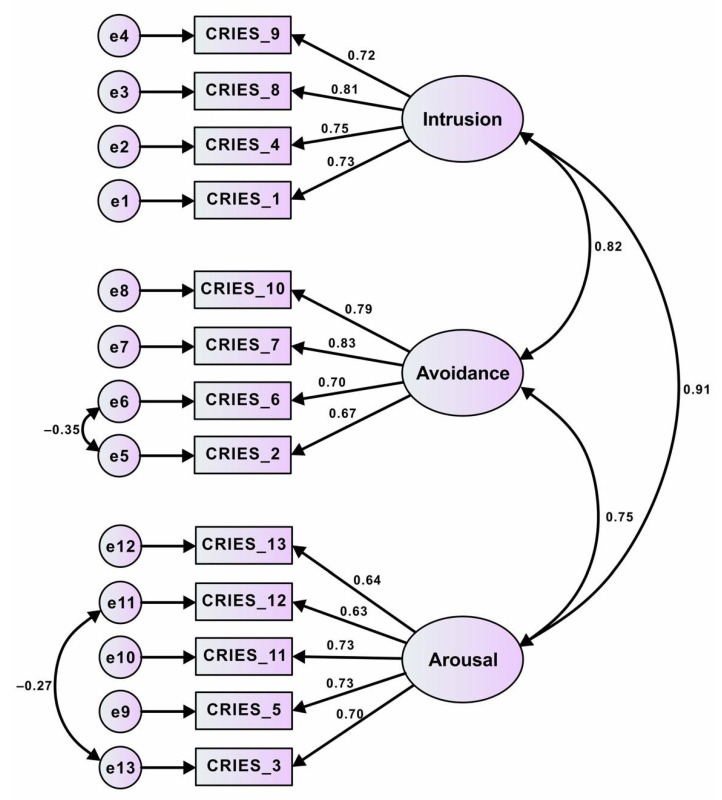
Three-structure model of Children’s Revised Impact of Event Scale 13 (CRIES-13) for all participants (adolescents).

The hypothesized three-factor structure of the CRIES-13 exhibited a good fit for the collected data because all the recommended criteria were met. The initial results are as follows: *x*^2^/*df* = 3.449, RMSEA = 0.084, GFI = 0.913, CFI = 0.931, TLI = 0.913, and SRMR = 0.049. Through the use of standardized estimates, our factor loadings were revealed to be very good (0.63–0.83) and significant. However, the evaluation of the modified indices suggested that residual covariances should be added for the error terms of items 2, 3, 6, and 12. The final results, which indicate a better model fit, are as follows: *x*^2^/*df* = 2.806, RMSEA = 0.072, GFI = 0.931, CFI = 0.951, TLI = 0.936, and SRMR = 0.040. The final model was used for further testing (Figure 1).

### 3.4. Measuring Invariance through MGCFA

The MGCFA results are presented in Table 2. Configural invariance analysis revealed that the model (M1) fit the data favorably (CFI = 0.922 and RMSEA = 0.064), and all factor loadings were significant (*p* < 0.05), indicating that the male and female groups were equivalent. The metric invariance model (M2) exhibited a good fit (CFI = 0.918 and RMSEA = 0.063). Significant changes occurred for ΔCFI (0.014) and ΔRMSEA (0.002), suggesting that metric invariance was achieved. Scalar analysis revealed that the model (M3) fit the data favorably (CFI = 0.908 and RMSEA = 0.066). The corresponding ΔCFI and ΔRMSEA were acceptable (0.010 and 0.002, respectively). For the highest level of invariance (i.e., full analysis), CFI = 0.895, and RMSEA = 0.066. The corresponding ΔCFI and ΔRMSEA were 0.013 and 0.000, respectively, indicating that invariance was achieved, although the model fit based on the CFI only failed to meet the criteria by a small margin (Table 2).

### 3.5. Reliability

Results of the reliability analysis are presented in Table 3. The coefficients for all 13 scale items were acceptable across the two gender groups (Cronbach’s alpha > 0.70); the recommended criteria were met. In addition, the obtained composite reliability values ranged from 0.81 to 0.84 (Table 3), indicating that their internal consistency was adequate (i.e., higher than the suggested level of > 0.70) [45].

## 4. Discussion

This study is the first to translate and evaluate the psychometric characteristics of the CRIES-13 in the context of Indonesian adolescents at risk of exposure to COVID-19. The scale, which was initially developed for predominantly English-speaking countries, should be developed to ensure that it can assess PTSD symptoms in non-English-speaking countries. Therefore, in the present study, a precise translation method was applied, and a team of experts were asked to confirm the scale’s validity such that it can be applied to a new population (i.e., Indonesian adolescents). All the items of the translated Indonesian scale had favorable content validity. The aforementioned evaluation method was required to verify the overall quality of the scale [32,33]. No study that has evaluated the CRIES-13 has applied this method; our study is the first to assess and verify the psychometric properties of the scale.

The three-factor structure of the original scale was also replicable for our study population. The translated Indonesian scale exhibited excellent construct validity, thereby validating the factor structure of the original scale [17]; this finding is consistent with those of other child and adolescent studies [18,19,20,22,23]. Our findings differ from those of other studies that examined similar age groups and confirmed a two-factor solution [24,25], in which the intrusion and arousal constructs were present in a single latent factor. In our study, intrusion, avoidance, and arousal were three correlated factor structures that formed the most suitable model for the CRIES-13.

Although the evaluation of the factor loadings of each item in our model suggested a good fit, we considered modifying our model by adding two residual covariances to increase its goodness of fit. This was based on several theoretical considerations. First, correlated errors were discovered between items 2 (“Do you try to remove it from your memory?”) and 6 (“Do you avoid reminders of it (e.g., places or situations)?”), which were both loaded onto the avoidance construct and represent attempts by an individual to eliminate trauma-inducing elements. Second, correlated errors were discovered between items 3 (“Do you have difficulties paying attention or concentrating?”) and 12 (“Are you alert and watchful even when there is no obvious need to be?”), which were both loaded onto the arousal construct; these two items reflect how an individual with arousal symptoms often enters a state of alertness because of an overactive fight-or-flight stress response, which often leads to decreased focus [14]. Deeba et al. also modified the CRIES-13 model to increase its fit [18] for the error terms of items 3 and 13, which were both loaded onto the arousal construct.

In our study, the lowest factor loading in the model was 0.63 (arousal factor, item 12: “Are you alert and watchful even when there is no obvious need to be?”). Although item 12 demonstrated a very good load [36], it had the lowest strength to measure arousal related to COVID-19 among the items. Item 12 was related to hypervigilance, indicating that when it was measured in situations of ongoing threat, its meaning might change [17,21]. Being alert is a natural reaction for individuals to have in the aftermath of a traumatic event because it directs them to be more aware of the event [47]. Adolescents in our study were alert to COVID-19 exposure, but their alert response was not as strong as it was in the early stages of the pandemic compared with Chinese adolescents, who reported higher alertness in the early stages of the pandemic [48].

The highest factor loading in our model was 0.83 (avoidance factor, item 7: “Do you try not to talk about it?”), indicating that it had the strongest association with the avoidance construct. A similar finding was also identified in a study involving Chinese adolescent survivors of an earthquake, which also found that item 7 exhibited excellent loading (0.75) on the avoidance construct [19]. In our study, adolescents’ responses to item 7 about the traumatic experiences of the COVID-19 pandemic were a precise variable to measure avoidance. Avoiding conversations about the traumatic event, people, or places that bring it to mind is an attempt to prevent upsetting memories, thoughts, or emotions related to the event [47].

In addition to factor loading, items in the arousal construct demonstrated relatively lower loading than the items in the intrusion and avoidance constructs. This result is similar to those reported by Deeba et al. [18] and Giannopoulou et al. [21]. Low correlations between arousal items and their respective factors in this study might be due to the fading effect of the adolescents’ current COVID-19 experience. Nevertheless, items in the arousal construct demonstrated very good to excellent relationships with the arousal construct, indicating that they were accurate variables to measure arousal responses toward a stressful event and important components of PTSD symptoms [47].

For measurement invariance, we achieved highly favorable results for configural, metric, scalar, and full invariance across gender groups. Our measurement invariance findings align with those of a study by Veronese and Pepe [23], who applied the CRIES-13 to a population of Palestinian male and female children and reported that the scale was partially invariant. In contrast to our study findings, Deeba et al. [18] reported that four-level invariance was not achieved across gender groups in a Bangladeshi population because their male participants tended to perceive their PTSD symptoms as being more severe relative to their female participants. Our research revealed that the structure of the translated Indonesian CRIES-13 was appropriate for evaluating comparable constructs and item content for both male and female adolescents. Therefore, the effect of gender on the design of the Indonesian CRIES-13 version can be disregarded in future studies that examine both male and female individuals.

For reliability, our findings pertaining to Cronbach’s alpha and composite reliability coefficients verified the consistency of each construct for each gender group and the overall sample. Although correlated errors were discovered among the items in our model, the results for both types of coefficients were similar [49]. These findings indicate that the three constructs exhibited high reliability and are comparable to the findings of the studies that examined children and adolescents exposed to earthquakes in Greece [21] and Bangladesh [18]. Because the Cronbach’s alpha and composite reliability values obtained were >0.7, the scale examined in the present study can be regarded as a legitimate and reliable tool for measuring the PTSD symptoms exhibited by adolescents at risk of exposure to traumatic events. A high composite reliability indicates that the items of a scale are reliable and exhibit high correlations when they correspond to the same construct [45].

Although our findings validate the original version of the CRIES-13, the present study has several limitations. First, we enrolled Indonesian junior high school students who experienced a specific traumatic event; our results may not be generalizable to other adolescent populations or to individuals who have experienced other types of traumatic events. However, our findings help clarify how PTSD symptoms are experienced by people with a non-English-speaking background. Second, because the data collection process was conducted online, we could not directly contact the adolescents and make observations to accurately determine the average time they took to complete the survey. Third, because this was a cross-sectional study, we cannot determine whether our findings are generalizable to other time periods. Nevertheless, our study contributes to the body of knowledge pertaining to the CRIES-13. For instance, measurement invariance was assessed in our study through the MGCFA, which is a component used to identify score validity evidence and assess construct-irrelevant variance [44]. To perform meaningful comparisons of CRIES-13 item interpretations and its subscales across specific groups, evidence must be gathered to support theoretical constructs and measurement invariance.

## 5. Conclusions

In conclusion, the use of the CRIES-13 is strongly supported by our findings. The present study revealed that the translated Indonesian CRIES-13 has distinct psychometric properties representing satisfactory content validity, a good three-factor structure that replicates the original version of the scale, and adequate internal consistency and reliability; researchers can confidently consider this scale as qualified. The measurement of PTSD symptoms may vary depending on the social networks and cultural backgrounds of a given community. However, posttraumatic stress experiences are not culture bound, and the CRIES-13 can measure different responses to trauma accurately. Researchers will benefit considerably from the availability of the translated Indonesian CRIES-13 as a robust PTSD scale. To clarify various characteristics and increase the diversity of scale assessment, further research involving the use of this scale should be conducted.

## Figures and Tables

**Table 1:** Participant demographic characteristics (*n* = 346).

Variable	*n*	%
Age (years)		
12–13	175	50.58
14–15	171	49.42
Gender		
Male	144	41.60
Female	202	68.90
Ethnicity		
Javanese	317	91.60
Other	29	8.40
Grade level		
7th	79	22.80
8th	267	77.20
Religion		
Muslim	293	84.70
Non-Muslim	53	15.30
Living arrangement		
Living with parents	301	87.00
Living with others	45	13.00
Home quarantine		
Yes	5	1.40
No	341	98.60

Note: The mean ± standard deviation for the participants’ age is 13.56 ± 0.786.

**Table 2:** Model fit of CRIES-13.

Model	*x* ^2^	*df*	*x* ^2^ */df*	CFI	RMSEA	Comparison	Δ*x*^2^	Δ *df*	ΔCFI	ΔRMSEA
M0	168.368	60	2.806	0.951	0.072					
M1	291.667	120	2.431	0.922	0.064					
M2	310.198	130	2.386	0.918	0.063	M2 vs. M1	18.531	10	0.014	0.001
M3	337.986	136	2.485	0.908	0.066	M3 vs. M2	27.788	6	0.010	0.002
M4	380.232	151	2.518	0.895	0.066	M4 vs. M3	42.246	15	0.013	0.000

Abbreviations: CRIES-13, Children’s Revised Impact of Event Scale 13; *x*^2^*/df*, ratio of chi-square to degrees of freedom; CFI, comparative fit index; RMSEA, root mean square error of approximation; M0, initial model; M1, configural invariance model; M2, metric invariance model; M3, scalar invariance model; M4, full invariance model.

**Table 3:** Scale description and internal consistency reliability of CRIES-13.

Item Number and Description	Mean	SD	Cronbach’s Alpha			Composite Reliability		
			Overall	Male	Female	Overall	Male	Female
**Intrusion**	4.27	4.81	0.83	0.79	0.85	0.84	0.80	0.86
Do you think about it even when you don’t mean to?	1.32	1.52						
4. Do you have waves of strong feelings about it?	0.95	1.48						
8. Do images of it pop into your mind?	1.20	1.54						
9. Do other things keep making you think about it?	0.80	1.33						
**Avoidance**	5.98	5.82	0.82	0.81	0.82	0.83	0.82	0.83
2. Do you try to forget about it?	1.75	1.88						
6. Do you avoid reminders of it (e.g., places or situations)?	1.10	1.59						
7. Do you try not to talk about it?	1.36	1.83						
10. Do you try not to think about it?	1.77	1.92						
**Arousal**	5.45	5.72	0.80	0.71	0.83	0.812	0.72	0.84
3. Do you have difficulty paying attention or concentrating?	0.91	1.37						
5. Do you get startled more easily or feel more nervous than you did before it happened?	0.86	1.46						
11. Are you easily irritated?	1.20	1.55						
12. Are you alert and watchful even when there is no obvious need to be?	1.73	1.80						
13. Do you have trouble sleeping?	0.75	1.43						
**Total score**	15.70	14.62	0.91					

Abbreviations: CRIES-13, Children’s Revised Impact of Event Scale 13; SD, standard deviation.

## Data Availability

The data presented in this study are available upon request from the corresponding author.

## Supplementary Materials

The following supporting information can be downloaded at: https://www.mdpi.com/article/10.3390/ijerph192417069/s1, Table S1: Content validity ratings for Children’s Revised Impact of Event Scale 13 (CRIES-13).
