# Supplementary material for: Psychometric Validation of the Indonesian Version of Children’s Revised Impact of Event Scale 13
Source: Int J Environ Res Public Health. 2022 Dec 19;19(24):17069. doi: 10.3390/ijerph192417069 (PMC9778705; doi:10.3390/ijerph192417069)
Supplement: Supplementary file 1 [file ijerph-19-17069-s001.zip › ijerph-2063237-supplementary.pdf]

Supplementary Table S1. Content validity ratings for CRIES-13.

Items	Expert 1	Expert 2	Expert 3	Expert 4	Expert 5	Expert 6	Experts in agreement	I-CVI	UA
1	1	1	1	1	1	1	6	1	1
2	1	1	1	1	1	1	6	1	1
3	1	1	1	1	1	1	6	1	1
4	1	1	1	1	1	1	6	1	1
5	1	1	1	1	1	1	6	1	1
6	1	1	1	1	1	1	6	1	1
7	1	1	1	1	1	1	6	1	1
8	1	1	1	1	1	1	6	1	1
9	1	0	1	1	1	1	5	0.83	0
10	1	1	1	1	1	1	6	1	1
11	1	1	1	1	1	1	6	1	1
12	1	1	1	1	1	1	6	1	1
13	1	1	1	1	1	1	6	1	1
**S-CVI/Ave**								0.99	
**S-CVI/UA**									0.92
**Proportional relevance**	1	0.92	1	1	1	1			
**Average proportion of items**							0.99		

Abbreviations: CRIES-13, Children's Revised Impact of Event Scale 13; I-CVI, item-level content validity index; S-CVI, scale-level content validity index; UA, universal agreement; S-CVI/Ave, scale-level content validity index based on averaging; S-CVI/UA, scale-level content validity index based on universal agreement.
